# A Rare Presentation of Brodie Abscess in the Clavicle

**DOI:** 10.5435/JAAOSGlobal-D-20-00249

**Published:** 2021-04-13

**Authors:** Thomas B. Lynch, Ryan Siu, Taylor Bates, Rachel A. Cuenca

**Affiliations:** From the San Antonio Military Medical Center (Dr. Lynch, Dr. Bates, and Dr. Cuenca), Fort Sam Houston, TX, and the California Northstate University College of Medicine (Siu), Elk Grove, CA.

## Abstract

A 12-year-old otherwise healthy boy presented with acute shoulder pain and remote history of trauma. Despite an unimpressive clinical examination, laboratory workup, and initial radiographic evaluation, the patient was ultimately diagnosed with a Brodie abscess of the distal clavicle. Complete resolution was achieved with débridement and tailored antibiotic therapy. These abscesses are rare, often presenting surreptitiously with nonspecific symptoms and without systemic signs of infection. Therefore, maintaining a broad differential and high clinical suspicion is crucial to mitigate the increased morbidity that can result from a delayed diagnosis.

In 1832, Sir Benjamin Collins Brodie first described a chronic inflammatory condition affecting the tibia after performing a lower extremity amputation on a patient with over a decade of leg pain and swelling. Examination of the amputated specimen revealed a walnut sized collection of pus, leading him to the conclusion that trephination and abscess decompression should be the cornerstone of treatment. Brodie^1^ abscess thus describes a subset of subacute osteomyelitis with an accompanied intraosseous collection of purulence. Bacterial seeding can occur from a variety of mechanisms to include penetrating trauma, spread of infection from adjacent soft tissues or joints, or hematogenous dissemination.^2^ In subacute hematogenous osteomyelitis, the proposed mechanism involves transient bacteremia from otitis media, pharyngitis, or other innocuous activities, such as a dental cleaning.^3^ These infections are often subtle and often go undiagnosed for long periods of time.

The most commonly described locations for hematogenous osteomyelitis are the metaphyseal regions of long bones. This is likely because of the slow blood flow within the serpentine vasculature, which predisposes these sites to the accumulation of bacterial pathogens.^2,4^ In addition, trauma may predispose to infection because of bacterial seeding of the hematoma. Malignancy must also be considered because Brodie abscesses can have a similar presentation to other disease processes, including a variety of musculoskeletal tumors.^5,6,7,8^

We present a case of a Brodie abscess in a 12-year-old boy after a presumed occult distal clavicle fracture. Possible seeding events include an upper respiratory infection, dental cleaning, and remote trauma. The treatment was comprised of surgical débridement followed by a 6-week course of tailored antibiotic therapy. The ambiguous nature of these infections can result in a delayed diagnosis or misdiagnosis with risk of increased morbidity. Providers must have a high index of suspicion and broad differential for patients with a remote history of trauma and focal pain despite normal examination findings.

## Case Report

A 12-year-old boy presented to the emergency department with chief complaint of 2 days of shoulder pain. His history included a seven-foot fall onto his shoulder 4 weeks before presentation. He returned to full activities after a short period of conservative management. In addition to the shoulder injury, he reported a subjective fever and symptoms suggestive of an upper respiratory tract infection 2 weeks earlier and had undergone a dental cleaning 1 week before presentation.

The patient's medical and surgical histories were normal. On presentation, he was afebrile (Tmax of 99.1°F) with normal vital signs. Shoulder examination revealed intact skin with point tenderness of the distal clavicle and overlying erythema but no fluctuance. His examination was otherwise normal. Laboratory studies included a c-reactive protein of 0.6 mg/L (nmL < 1.0), erythrocyte sedimentation rate of 28 mm/hr (nmL < 20), and white blood cell count of 11.1 × 10^3^/µL (4.5 to 11.5).

**Figure 1:**
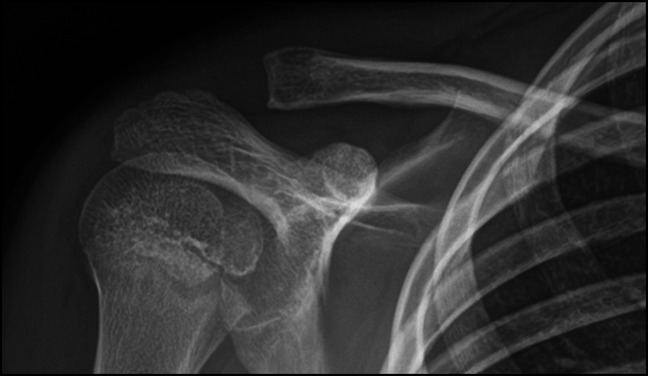
Anteroposterior radiograph of the right shoulder demonstrating a lytic lesion with cortical thinning affecting the distal clavicle.

**Figure 2:**
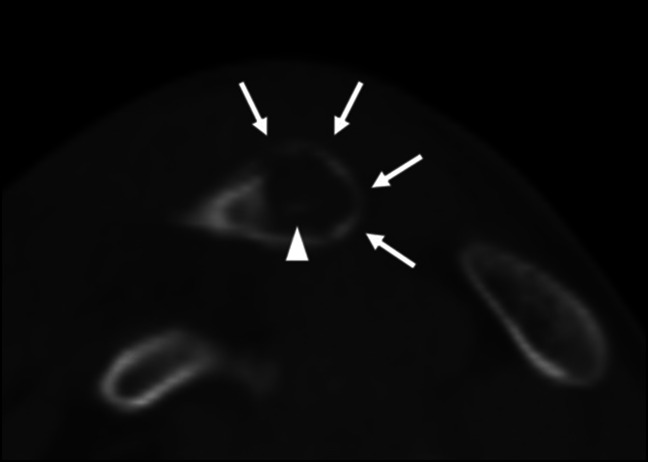
Sagittal CT (2 mm cuts) of the clavicle demonstrating cortical erosion (arrows) with an intramedullary sequestrum (arrowhead).

**Figure 3:**
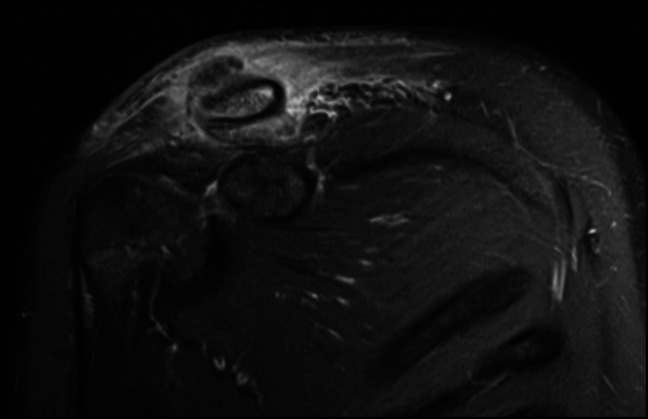
Coronal postcontrast T1-weighted MRI periosteal reaction with cortical erosion, periosteal elevation, and associated soft-tissue swelling.

**Figure 4:**
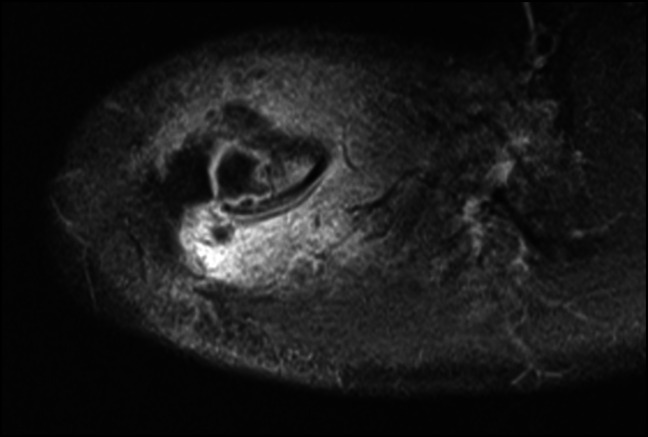
Axial postcontrast T1-weighted MRI demonstrating penumbra sign with cloaca.

Plain radiographs of the right shoulder revealed a small lytic lesion about the distal clavicle (Figure 1). To further characterize the lytic lesion, both noncontrast CT scan and MRI with and without contrast were done. An intramedullary cortical erosion with sequestrum was discovered on CT imaging (Figure 2). MRI revealed periosteal elevation with a fluid collection about the superior distal clavicle and adjacent soft-tissue swelling (Figure 3). T1-weighted imaging showed a penumbra sign (Figure 4).

**Figure 5:**
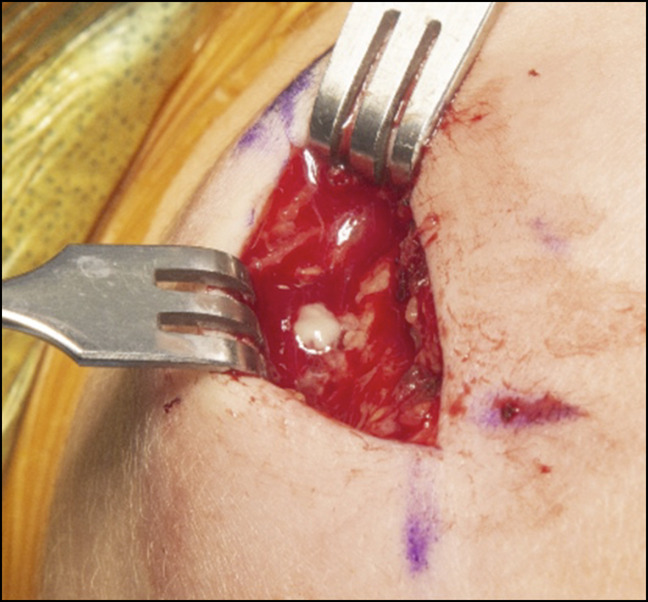
Intraoperative photograph of the distal clavicle demonstrating frank purulence after abscess decompression.

**Figure 6:**
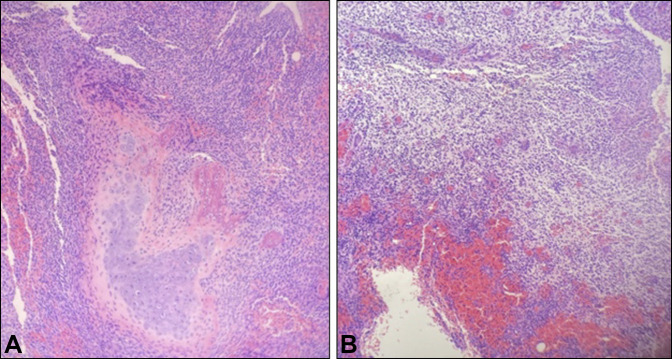
**A** and **B**, Histological specimens after hematoxylin-eosin staining demonstrating neutrophilic and lymphocytic inflammation with reactive new bone formation.

Given the clinical and imaging findings, the patient was indicated for surgical débridement of osteomyelitis with an associated abscess. Purulence was encountered during surgical exposure, which was evacuated with bony curettage (Figure 5). Histologic specimens collected at the time of débridement revealed neutrophilic and lymphocytic infiltration with reactive bone formation (Figure 6, A and B). Cultures grew methicillin sensitive *Staphylococcus aureus*. The patient was started on broad spectrum antibiotics postoperatively followed by a 6-week course of cephalexin based on the results of bacterial sensitivity testing.

Postoperatively, the patient's surgical site healed without complication, and he completed the antibiotic regiment as prescribed. At 10 months postoperatively, he had full range of motion with no pain and has returned to full activity.

## Discussion

We present the first confirmed report of a clavicular Brodie abscess. This case highlights the importance of correlating patient history, physical examination, and imaging findings to make an accurate diagnosis. Furthermore, it describes an atypical location for abscess formation. van der Naald et al^9^ did a systematic review of over 400 reported Brodie abscesses. The authors found that about 50% develop in the tibia and just over 30% in the femur. Despite this large series, no cases involving the clavicle were identified. Lopes et al^10^ did a quantitative analysis of the plain radiographic appearance of Brodie abscesses. In their series, the authors reported one suspected case affecting the clavicle; however, this was never confirmed.

Pediatric osteomyelitis of the clavicle is a rare condition and most commonly occurs through hematogenous spread.^11^ The mechanism of infection in this patient is likely an occult fracture of the distal clavicle causing a vascular disruption and hematoma, predisposing him to hematogenous seeding. This is similar to the slow flow vascular environment in the metaphyseal region of pediatric long bones where cases of osteomyelitis are most commonly found.^12^ Seeding bacteremia can arise from a variety of sources to include mucosal injuries from nasal bleeding, dental hygiene, chewing of hard foods, or defecation.^13^ This patient reported a recent dental cleaning and upper respiratory infection in the period after his injury and before worsening shoulder pain. Either could have been the cause of hematogenous seeding.

After bacterial seeding of the bone, a Brodie abscess develops when there is an inflammatory response with a collection of intramedullary purulence and vascular congestion. This results in cortical destruction with reactive bone formation that walls off the abscess (Figure 2).^13^ Recent upper respiratory infection and a remote history of minor trauma are two independent risk factors commonly reported to be associated with Brodie abscess.^14^ van der Naald et al^9^ found that 25/56 cases reported a history of remote trauma, whereas 12/56 had a history of recent infection. In this case, there was both a history of trauma and recent upper respiratory infection.

Patients with osteomyelitis commonly present without signs of systemic infection and normal inflammatory markers.^2,14,15^ Within the first 2 weeks of symptom onset, 80% of radiographs are negative for osteomyelitis.^15^ When osteomyelitis is present on radiographs, periosteal elevation, soft-tissue swelling, and a focal lytic lesion may be seen.^12^ In this patient, radiographs were initially interpreted as negative by multiple providers (Figure 1). However, a focal lytic lesion of the distal clavicle was noticed on correlation with examination. Advanced imaging was used to better characterize the lesion. Differentiating a Brodie abscess from other pathology can be difficult because it can mimic osteoid osteoma, chondrosarcoma, and eosinophilic granuloma and requires advanced imaging.^5,6,7,8^

MRI is critical to discern osteomyelitis from other potential diagnoses. Given the differential at the time of MRI administration included infection versus bone tumor noncontrast and contrast enhanced MRI was done. Noncontrast sequences are useful in evaluating the degree of medullary extension. The “penumbra” sign describes the presence of a T1 hypointense center with a surrounding hyperintense T1 signal (Figure 4).^16^ This finding that has been shown to be a reliably specific finding in the diagnosis osteomyelitis in comparison with other potential bone tumors. Shimose et al^17^ published a series of 244 patients with concern for osteomyelitis versus bone tumors and found the specificity of the penumbra sign for osteomyelitis was 99.1% specific for osteomyelitis. Although CT provided better characterization of the osseous abnormality, it was ultimately not a critical step in arriving at the correct diagnosis and treatment plan.

*Staphylococcus aureus* accounts for most of osteomyelitis cases. Other common pathogens include coagulase-negative staphylococcus, beta hemolytic streptococcus, enterococci, Gram-negative bacilli, and anaerobes.^3,14,18^ *Salmonella* species should be considered in patients with sickle cell disease. Treatment of acute or subacute osteomyelitis is tailored to the sensitivities of the offending pathogen. There is no consensus regarding the guidelines for the duration of antibiotic therapy. Some recommend courses of parenteral antibiotics for greater than 7 days, followed by oral antibiotics, whereas others advocate expedited transition from parental antibiotics.^14,19^ In a review of cohort studies, Le Saux et al^20^ reported that children treated with 7 days or less of parenteral antibiotics had cure rates surpassing 90%. The duration of parenteral medication is dependent on patient improvement because more resistant pathogens may require prolonged therapy. After administration of parenteral antimicrobials, individualized oral therapy is recommended for 4 to 6 weeks.^14,18,20,21^

A high index of suspicion is required because patients presenting with subacute hematogenous osteomyelitis may have subtle clinical features. To reduce the incidence of serious sequelae, a swift diagnosis and initiation of treatment is necessary.
